# Crystallization Products of Risedronate with Carbohydrates and Their Substituted Derivatives ^†^

**DOI:** 10.3390/molecules16053740

**Published:** 2011-05-04

**Authors:** Jiri Kos, Monika Pentakova, Zbynek Oktabec, Lukas Krejcik, Zuzana Mandelova, Pavla Harokova, Jana Hruskova, Tomas Pekarek, Ondrej Dammer, Marcela Tkadlecova, Jaroslav Havlicek, Jarmila Vinsova, Vladimir Kral, Jiri Dohnal, Josef Jampílek

**Affiliations:** 1 Faculty of Pharmacy, University of Veterinary and Pharmaceutical Sciences, Palackeho 1/3, 612 42 Brno, Czech Republic; 2 Zentiva k.s., U Kabelovny 130, 102 37 Prague 10, Czech Republic; 3 Faculty of Pharmacy in Hradec Kralove, Charles University in Prague, Heyrovskeho 1203, 500 05 Hradec Kralove, Czech Republic; 4 Faculty of Chemical Engineering, Institute of Chemical Technology, Technicka 5, 16628 Prague 6, Czech Republic

**Keywords:** risedronate, phenyl-β-D-galactopyranoside, polymorph P, FT-NIR, FT-Raman, CP/MAS NMR, XRPD, DSC

## Abstract

The gastrointestinal absorption of bisphosphonates is in general only about 1%. To address this problem mixtures of risedronate monosodium salt with twelve varied sugar alcohols, furanoses, pyranoses and eight gluco-, manno- and galactopyranoside derivatives as counterions were designed in an effort to prepare co-crystals/new entities with improved intestinal absorption. Crystalline forms were generated by means of kinetically and/or thermodynamically controlled crystallization processes. One hundred and fifty-two prepared samples were screened by means of FT-NIR and FT-Raman spectroscopy. No co-crystal was prepared, but noteworthy results were obtained. A new solid phase of risedronate monosodium salt generated in the presence of phenyl-β-D-galactopyranoside under thermodynamically controlled crystallization conditions was found and also characterized using solid state NMR spectroscopy, X-ray powder diffraction and differential scanning calorimetry. This new polymorph was named as form P. Interactions between risedronate monosodium salt and both carbohydrates were confirmed by means of molecular dynamics simulation. In the present study the relationships between the chemical structures of the studied compounds required for crystalline form change are discussed.

## 1. Introduction

Polymorphism of active pharmaceutical ingredients (APIs) is receiving increasing attention as an important physico-chemical parameter influencing bioavailability and stability of APIs and pharmaceuticals. Co-crystals of an API with common pharmaceutical excipients become very important as a tool to tune solubility and absorption. The application of co-crystal technologies has only recently been recognised as a way to enhance solubility, stability and the intellectual property (IP) position with respect to the development of active pharmaceutical ingredients. Unlike salt formation, co-crystallisation does not rely on ionisation of the API and the counterion to make a solid. Instead, both components utilise prominent intermolecular interactions, such as hydrogen bonding, to combine and yield a uniform crystalline material. Combining an API with a pharmaceutically acceptable agent in this guest/host manner has become an increasingly attractive route for developing pharmaceutical products. For example, co-crystallisation offers an alternative when salt screening is either unsuccessful or impossible (due to lack of ionisation sites) to improve the physical properties of a drug. Furthermore, exploring the co-crystallisation potential around an API increases the intellectual property protection over a particular drug product, thus reducing the risk of costly litigation and market erosion. A recent development in the field has not only shown co-crystallisation as an alternative to salt studies, but has also shown its combination with salts to yield co-crystals of salts [1]. Co-crystals of APIs with common pharmaceutical excipients are thus becoming very important [2,3].

Bisphosphonates (BPs) are the most widely used and the most effective bone resorption inhibitors currently available for treatment of Paget’s disease, tumour-associated bone disease and osteoporosis. All BPs have high affinity for bone mineral as a consequence of their P-C-P backbone structure, which allows chelation of calcium ions [4]. Following release from bone mineral during acidification by osteoclasts, BPs appear to be internalized specifically by osteoclasts, but not other bone cells [5]. The intracellular accumulation of BP leads to inhibition of osteoclast function due to changes in the cytoskeleton, loss of the ruffled border [5,6] and apoptosis [7,8,9,10]. The ability of BPs to inhibit bone resorption depends on the presence of two phosphonate groups in the P-C-P structure, which appears to be required for interaction with a molecular target in the osteoclast as well as for binding bone mineral [11,12,13].

**Figure 1:**
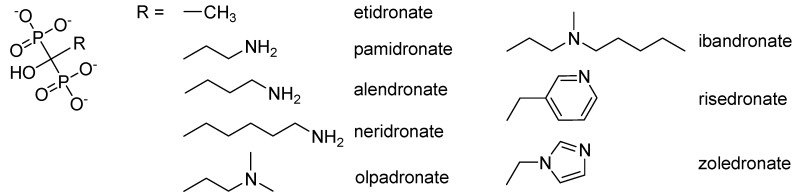
Structures of bisphosphonates used in practice.

Bisphosphonates such as the pyrophosphate analogues (see a general structure in Figure 1) are a group of drugs that are widely used in practice. There are several injectable bisphosphonates: etidronate (Didronel^®^), pamidronate (Aredia^®^) and zoledronate (Zometa^®^), which may be administered every three months or yearly. Peroral bisphosphonates alendronate (Fosamax^®^) and risedronate (Actonel^®^, Risendros^®^) are taken daily, weekly or monthly, and ibandronate (Boniva^®^) is approved to be taken monthly. Risedronate has a chemically unique component as compared with other bisphosphonates, which is believed to reduce the likelihood of gastro-intestinal side effects. Risedronate is more potent in blocking the dissolution of bone than etidronate and alendronate. [14,15]. Oral bioavailability of these bisphosphonates is very low (their gastrointestinal absorption is about 1%) due to their high hydrophilicity [16].

In general, the following structural modifications are the best way to improve permeability: (*i*) replacement of ionisable groups by non-ionizable groups; (*ii*) increase of lipophilicity; (*iii*) isosteric replacement of polar groups; (*iv*) esterification of carboxylic acid; (*v*) reduction of hydrogen bonding and polarity; (*vi*) reduction of size; (*vii*) addition of a nonpolar side chain; (*viii*) preparation of prodrugs. Generally these strategies are based on a few fundamental concepts: reduction of ionizability, increase of lipophilicity, reduction of polarity or reduction of hydrogen bond donors or acceptors. Thus, it is important to assess permeability early and to build permeability improvement into the synthetic plan from the beginning. This could rescue a chemical series that has great potential and improve drug exposure in animal pharmacology and pharmacokinetic studies. Formulation is other strategy for improving permeability and bioavailability. For example, permeability enhancers, surfactants or pharmaceutical complexing agents can be used in the oral dosage form [17].

Due to the above mentioned facts, the aim of this investigation was to design various mixtures of a bisphosphonate and carbohydrates in an effort to prepare co-crystals/new entities of risedronate with higher bioavailability. In the present study various mixtures of risedronate and carbohydrates (as excipients) in different ratios and under various conditions were prepared. All the prepared mixtures (solid compounds) were characterized by means of the Fourier Transform Near-Infrared (FT-NIR) spectroscopy [18]. Potential new entities were also characterized by means of FT-Raman spectroscopy, solid-state NMR spectroscopy, X-Ray Powder Diffraction (XRPD) and Differential Scanning Calorimetry (DSC). The confirmed potential co-crystals would be investigated for their absorption by means of experiments using the Parallel Artificial Membrane Permeation Assay (PAMPA, http://www.bdbeurope.com) [17]. This is a follow-up paper to our previous works [19,20,21,22] dealing with preparation and characterization of new crystalline forms and/or potential co-crystals of APIs with excipients.

## 2. Results and Discussion

### 2.1. Chemistry

**Figure 2:**
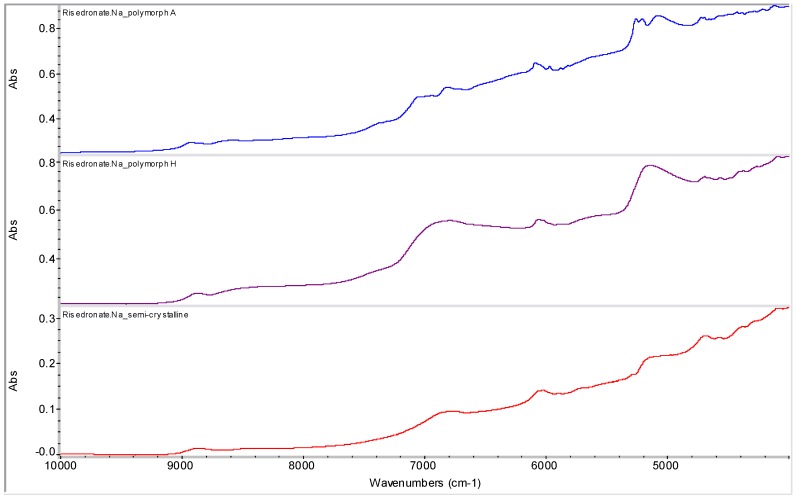
NIR spectra of risedronate mono-sodium salt polymorph A, polymorph H and starting semi-crystalline risedronate monosodium salt.

Semi-crystalline risedronate monosodium salt [sodium 1-hydroxy-1-phosphono-2-(pyridin-3-yl-ethyl)phosphonate, RSN] was used as a starting material [23]. It is a white powder, freely soluble in water, and practically insoluble in organic solvents. Nine different polymorphic and pseudo-polymorphic forms of sodium risedronate identified as A, B, B1, BB, D, E, F, G and H were described [24,25,26]. The crystal structures of four different hydrates (monohydrate, dihydrate, hemipentahydrate and variable hydrate) and an anhydrate of sodium risedronate have been elucidated and discussed by Redman-Furey *et al.* [27] and Gossman *et al.* [28]. The sodium hemipentahydrate, which is the marketed form A, is the most stable of all these forms at ambient conditions (298 K, 50% room humidity) [24]. Recently three new phases were found and named J, K and M [29]. NIR spectra of polymorphs A, H and semi-crystalline risedronate mono-sodium salt are shown in Figure 2.

**Figure 3:**
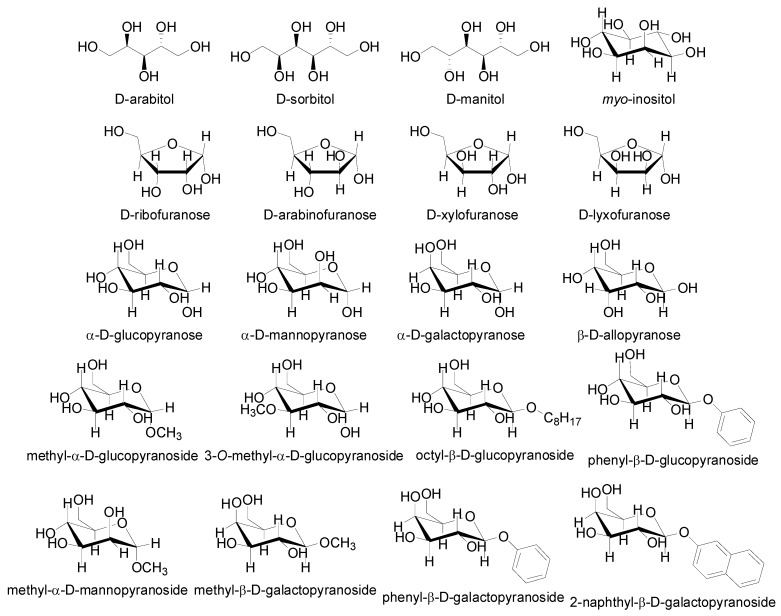
Structure of starting sugar alcohols, furanoses, pyranoses as well as gluco-, manno- and galactopyranoside derivatives used as potential counterions.

**Figure 4:**
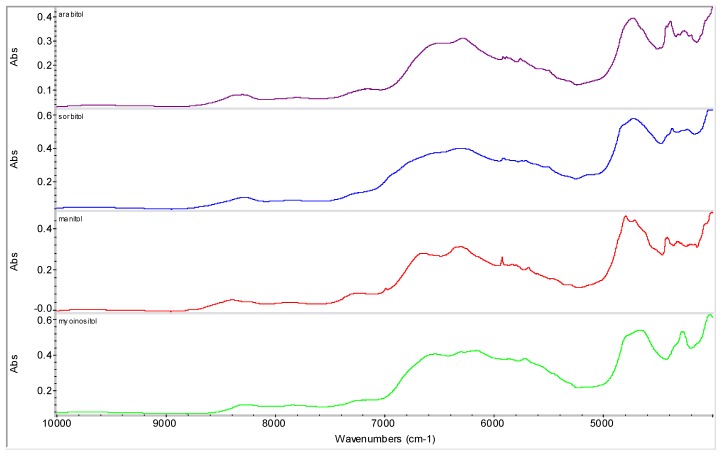
NIR spectra of discussed sugar alcohols.

**Figure 5:**
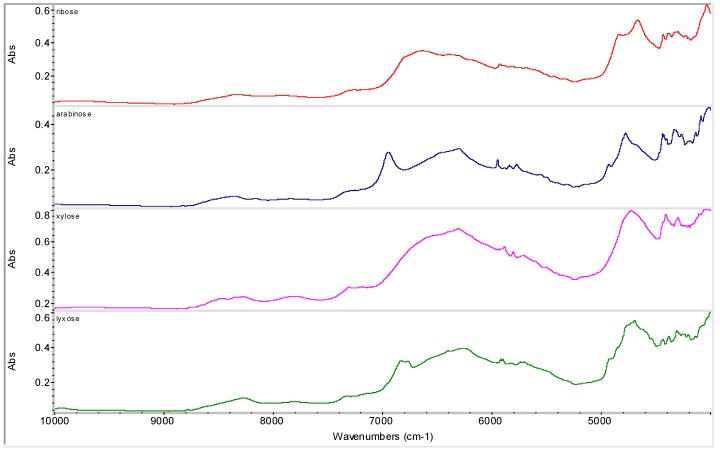
NIR spectra of discussed furanoses.

**Figure 6:**
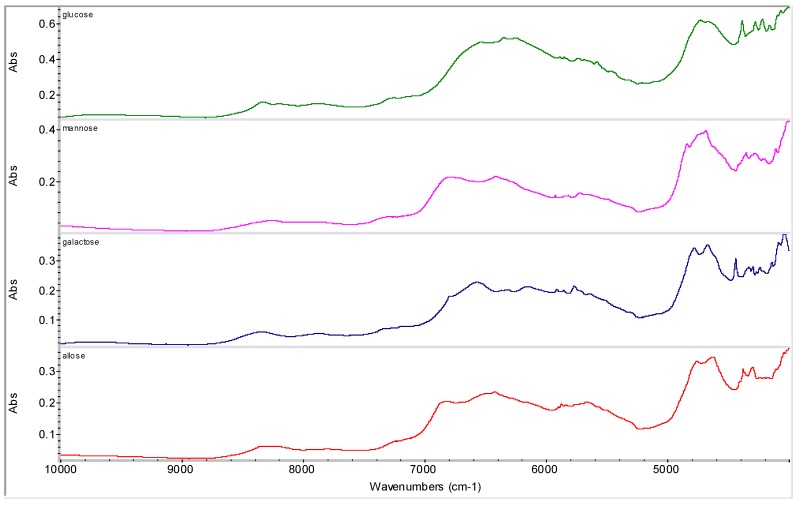
NIR spectra of discussed pyranoses.

**Figure 7:**
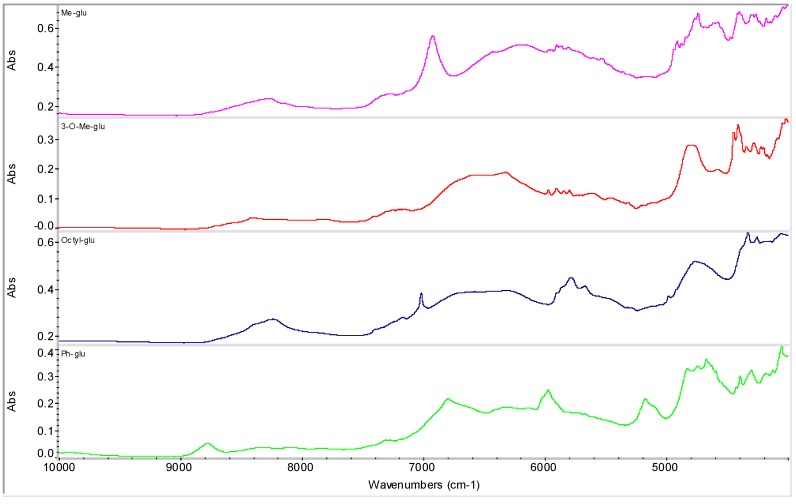
NIR spectra of D-glucopyranose derivatives. (methyl-α-D-glucopyranoside: Me-glu, 3-*O*-methyl-α-D-glucopyranoside: 3-*O*-Me-glu, octyl-β-D-glucopyranoside: Octyl-glu, phenyl-β-D-glucopyranoside: Ph-glu)

**Figure 8:**
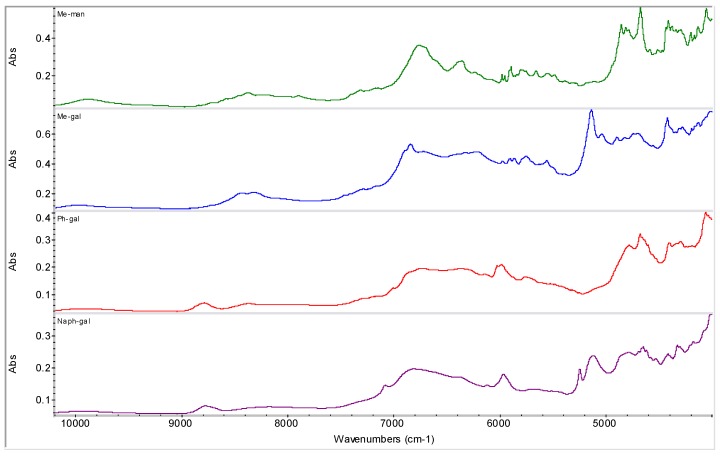
NIR spectra of D-manno- and D-galacopyranose derivatives. (methyl-α-D-mannopyranoside: Me-man, methyl-β-D-galactopyranoside: Me-gal, phenyl-β-D-galactopyranoside: Ph-gal, 2-naphthyl-β-D-galactopyranoside: Naph-gal)

Various sugar derivatives were evaluated as potential counterions: D-arabitol, D-sorbitol, D-manitol and *myo*-inositol (Figure 3 and Figure 4), D-ribofuranose, D-arabinofuranose, D-xylofuranose and D-lyxofuranose (Figure 3 and Figure 5), α-D-glucopyranose, α-D-mannopyranose, α-D-galactopyranose and β-D-allopyranose (Figure 3 and Figure 6) and methyl-α-D-glucopyranoside, 3-*O*-methyl-α-D-gluco-pyranoside, octyl-β-D-glucopyranoside, phenyl-β-D-glucopyranoside, methyl-α-D-mannopyranoside, methyl-β-D-galactopyranoside, phenyl-β-D-galactopyranoside and 2-nyphthyl-β-D-galactopyranoside (Figure 3, Figure 7 and Figure 8).

The evaluated samples were prepared by means of dissolution of risedronate mono-sodium salt and carbohydrates and subsequent reverse obtaining of solid compounds that were primarily characterized using the FT-NIR spectroscopy (diffuse reflectance method, DRIFT).

**Figure 9:**
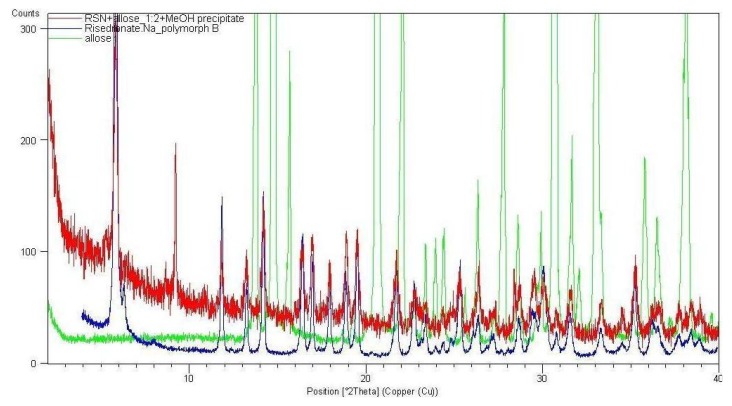
XRPD patterns of allose, sample RSN+allose in ratio 1:2 and risedronate mono-sodium salt form B.

From all tested agents only phenyl-β-D-galactopyranoside (Ph-gal) yielded interesting products with RSN. Other tested carbohydrates yielded either risedronate form A (in most cases), form H (in the case of the samples with *myo*-inositol, D-lyxofuranose, phenyl-β-D-glucopyranoside and naphthyl-β-D-galactopyranoside prepared by addition of MeOH and evaporation of liquid part at ambient temperature) or impure form B in the case of the sample with β-D-allopyranose (allose) precipitated by methanol, see Figure 9.

**Figure 10:**
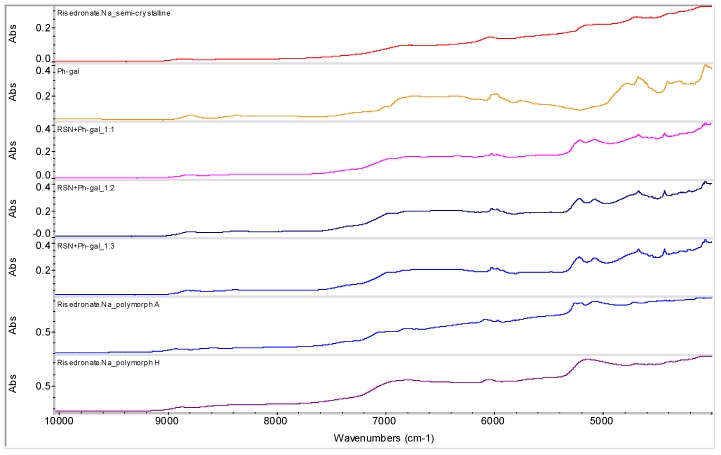
NIR spectra of semi-crystalline risedronate monosodium salt (RSN), forms A and H and phenyl-β-D-galactopyranoside (Ph-gal) and spectra of their mixtures in ratios 1:1, 1:2 and 1:3 prepared by evaporation at ambient temperature (samples **1**-**3**).

Samples of RSN+Ph-gal in ratios 1:1 (**1**), 1:2 (**2**) and 1:3 (**3**) were prepared by mixing saturated aqueous solutions and subsequent evaporation of water at ambient temperature. The spectra are illustrated in Figure 10. All three samples **1**-**3** contained risedronate polymorph A (the most thermodynamically stable risedronate form), see characteristic bands ranged 5,400–4,800 cm^−1^.

**Figure 11:**
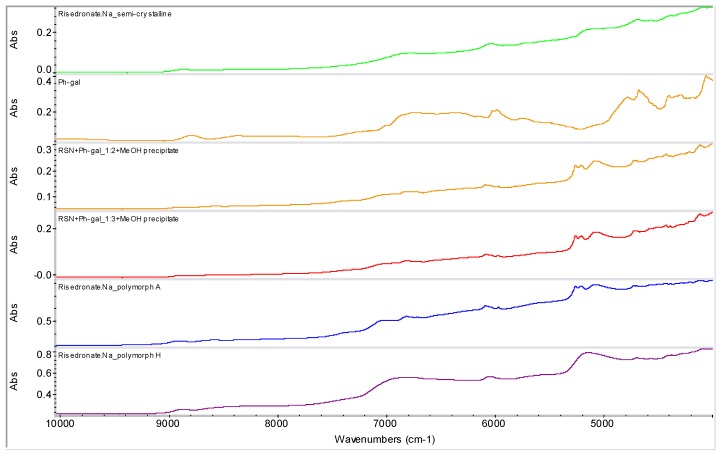
NIR spectra of semi-crystalline risedronate monosodium salt (RSN), forms A and H and phenyl-β-D-galactopyranoside (Ph-gal) and spectra of their mixtures in ratios 1:2 and 1:3 prepared by methanol precipitation (samples **4**, **5**).

Samples of RSN+Ph-gal in ratios 1:2 (**4**) and 1:3 (**5**) precipitated by methanol and filtered yielded again risedronate polymorph A (Figure 11), see characteristic bands ranged 5,400–4,800 cm^−1^. The NIR spectra of samples **1**-**5** seem to be very similar, see Figure 10 and Figure 11.

**Figure 12:**
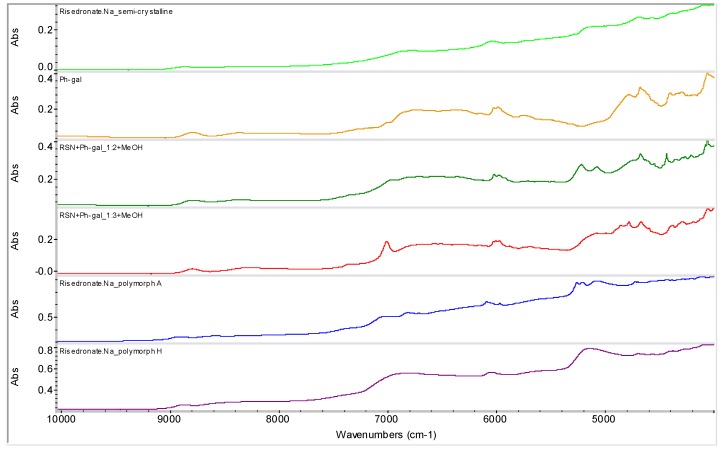
NIR spectra of semi-crystalline risedronate mono-sodium salt (RSN), forms A and H and phenyl-β-D-galactopyranoside (Ph-gal) and spectra of mixtures of RSN+Ph-gal in ratios 1:2 and 1:3 prepared by addition of MeOH and evaporation of liquid part at ambient temperature (samples **6**, **7**).

Figure 12 illustrates samples RSN+Ph-gal in ratios 1:2 (**6**) and 1:3 (**7**). The samples were generated by addition of MeOH and filtration of the obtained precipitate with following evaporation at ambient temperature. It is evident from this figure that sample **6** is absolutely different from all the above mentioned samples, the discussed risedronate sodium polymorphs and from sample **7**. Sample **7** seems to be a mixture of polymorphs. These facts were also confirmed by FT-Raman spectrometry and solid state NMR spectroscopy (see below). A change in the spectra of samples **6** and **7** can be observed in the range 7,100–4,900 cm^−1^. It can be concluded that the presence of phenyl-β-D-galactopyranoside and slow evaporation, *i.e.*, thermodynamically controlled crystallization process, with a small amount of methanol as anti-solvent provided risedronate in unknown forms.

**Figure 13:**
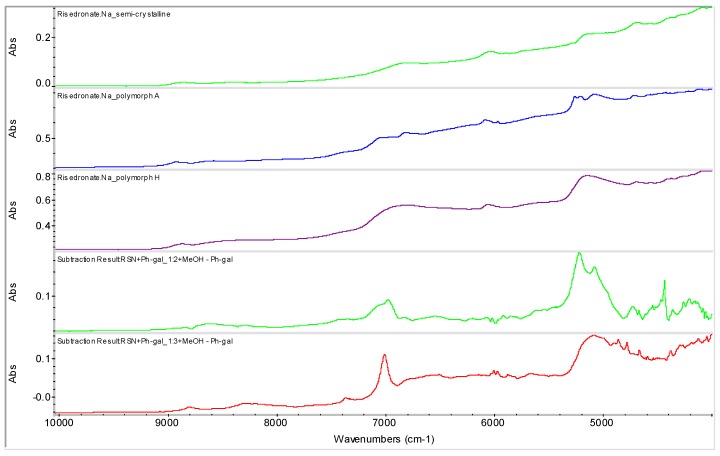
NIR spectra of semi-crystalline risedronate monosodium salt (RSN) and forms A and H and subtracted spectra of samples **6** and **7** (RSN+Ph-gal in ratios 1:2 and 1:3 prepared by addition of MeOH and evaporation of liquid part at ambient temperature).

The spectrum of phenyl-β-D-galactopyranoside was subtracted from the spectra of samples **6** and **7**, and the subtraction results are shown in Figure 13. On the basis of these subtracted spectra it can be concluded that the prepared sample **6** could be a new entity, because this subtracted result is different from the spectrum of the starting material, semi-crystalline risedronate monosodium salt and the stable risedronate forms A and H, *i.e.*, the final product is not a simple mixture of risedronate monosodium salt with phenyl-β-D-galactopyranoside.

**Figure 14:**
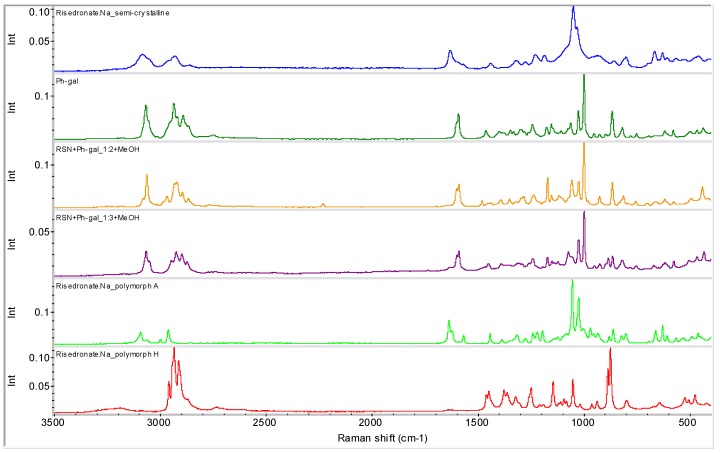
FT-Raman spectra of semi-crystalline risedronate monosodium salt (RSN), forms A and H, phenyl-β-D-galactopyranoside (Ph-gal) and samples **6** and **7**.

**Figure 15:**
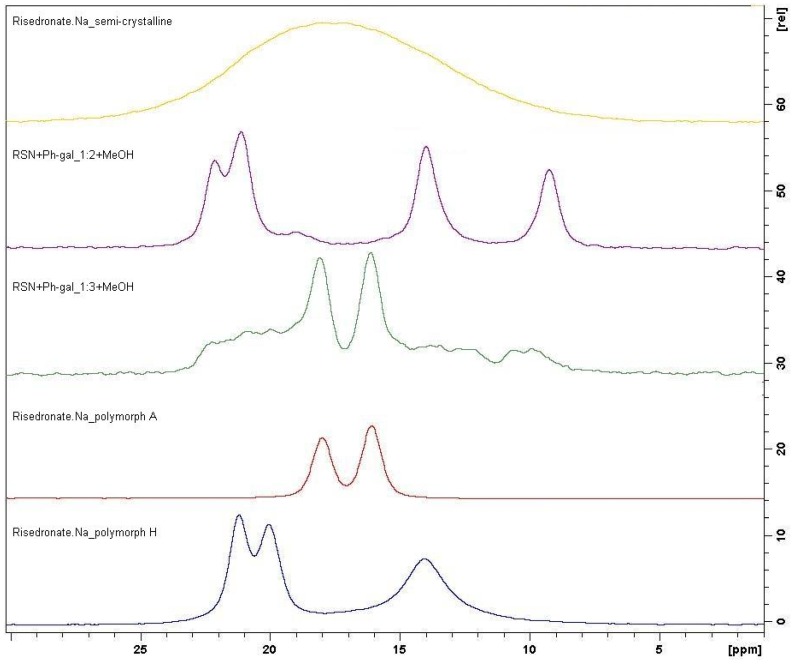
Comparison of ^31^P CP/MAS NMR spectra of semi-crystalline risedronate monosodium salt (RSN), forms A and H and samples **6** and **7**.

Samples **6** and **7** were also characterized by means of the Raman spectroscopy (see Figure 14) and ^31^P CP/MAS NMR spectroscopy (see Figure 15) for verification of the above mentioned hypothesis. Both methods confirmed the presence of a new entity in sample **6**.

Raman spectroscopy is more advantageous than middle IR spectroscopy due to lower wavenumbers observed – up to about 150 cm^−1^. Bands occurring at such low wavenumbers correspond to skeleton and lattice vibrations. Therefore non-covalent interactions are easier observable than in case of mid-IR (usually not lower than 400 cm^−1^).

**Figure 16:**
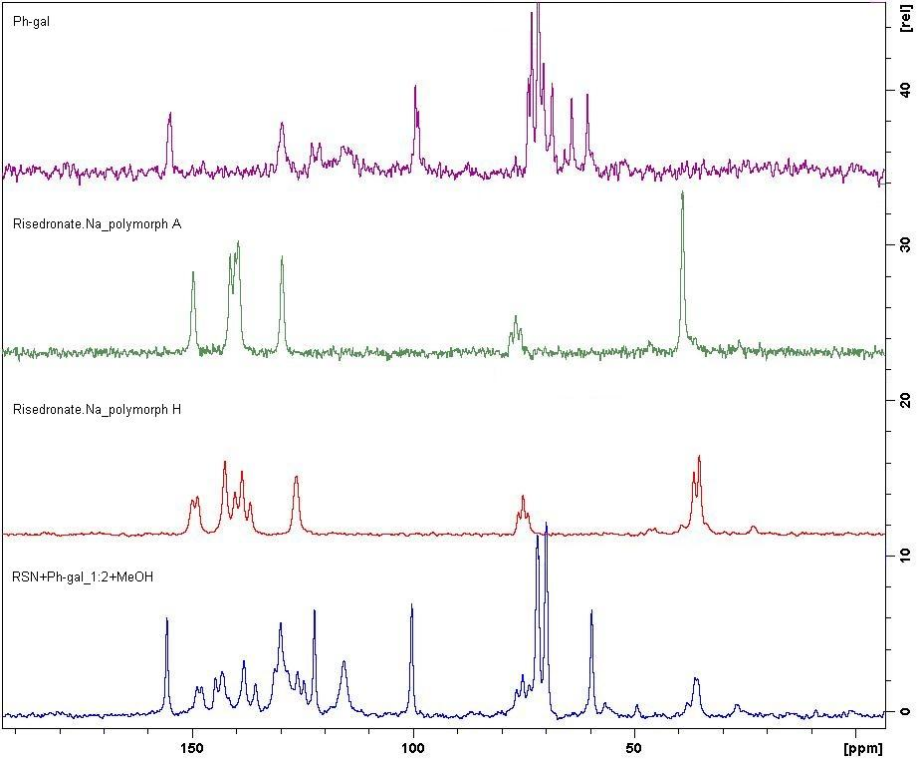
Comparison of ^13^C CP/MAS NMR spectra of semi-crystalline risedronate monosodium salt (RSN), forms A and H and phenyl-β-D-galactopyranoside (Ph-gal) and spectrum of potentially new crystalline form (sample **6**).

The ^31^P CP/MAS NMR method is much more sensitive and selective and this technique detected an interesting fact. Sample **7** (rate 1:3) seems to be a mixture of polymorph A and amorphous form of risedronate, while sample **6** (rate 1:2) seems to be absolutely different and it can be a potentially new crystalline form. This fact was also confirmed by means of the ^13^C CP/MAS NMR spectroscopy. Figure 16 presenting the respective results shows that sample **6** is not any mere simple mixture.

**Figure 17:**
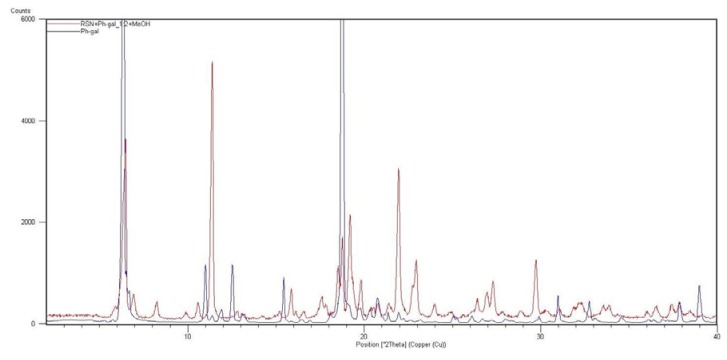
XRPD patterns of sample **6** (RSN+phenyl-D-galactopyranoside in ratio 1:2) and phenyl-β-D-galactopyranoside (Ph-gal).

Based on the above discussed results, sample **6** (RSN+Ph-gal in ratio 1:2) was additionally characterized by means of XRPD (see Figure 17) and also by DSC. An XRPD pattern corresponds to a crystalline sample. Visual comparison of the measured pattern with those published previously (namely forms A, B, BB, B1, C, D, E, F, G in WO 03/086355 [26] and forms J, K, M in Bruning *et al.* [29]) revealed that a new solid phase was formed. It is also supported by the absence of peaks of a co-crystal former (phenyl-β-D-galactopyranoside).

The DSC result confirmed the fact that phenylgalactopyranoside is not detectable in sample **6**. The DSC curve of phenyl-β-D-galactopyranoside showed an endotherm T_onset_ = 154.996 °C and T_peak_ = 155.897 °C. The DSC curve of the starting semi-crystalline risedronate monosodium salt showed an endotherm T_onset_ = 185.817 °C and T_peak_ = 236.968 °C. In the DSC curve of sample **6** the following endotherms can be observed: T_onset1_ = 84.560 °C and T_peak1_ = 86.181 °C; T_onset2_ = 115.535 °C and T_peak2_ = 121.260 °C; T_onset3_ = 144.944 °C and T_peak3_ = 149.444 °C.

All products of risedronate and β-D-allose in ratios 1:1, 1:2 and 1:3 generated by slow evaporation of water or water-MeOH mixture at ambient temperature contained the risedronate polymorphs A or H, thermodynamically stable risedronate forms. Based on this fact it can be concluded that the addition of MeOH as an anti-solvent is crucial for generation of a different entity. Rapid change of solubility equilibrium and fast precipitation (kinetically controlled crystallization process) of risedronate under the presence of β-D-allopyranose in excessive quantity caused generation of different form B, while slow evaporation, *i.e.*, thermodynamically controlled crystallization, led to preparation of stable polymorphs. β-D-Allose modifies the environment from which risedronate is crystallized, but it is not detectable in the final crystalline form (probably it is not present).

Different interactions of risedronate monosodium salt with β-D-allopyranose are probably caused by the opposite orientation of hydroxyl moiety in C_(3)_ in position 4 of the tetrahydropyran ring in comparison with α-D-gluco-, α-D-manno- and α-D-galactopyranose. The β-position of the hydroxyl moiety in C_(1)_ of β-D-allopyranose possesses also a *cis*-orientation with respect to the pyran oxygen in position 1 of the tetrahydropyran ring. As bonds influencing generating crystalline forms are formed by non-binding interactions (e.g., by *H*-bonds, ionic bonds, van der Waals forces (dispersion attractions, dipole-dipole, dipole-induced dipole interactions) and hydrophobic interactions), the steric arrangement of hydroxyl moieties on pyranose skeletons seems to be important for co-crystal generation.

Sugar alcohols did not provide any different forms or co-crystals with risedronate. The polyols used are acyclic compounds, or probably important heterocyclic oxygen is not present in the ring. In the case of *myo*-inositol, where only the different risedronate polymorph H was detected, *cis*-oriented hydroxyl moieties are in C_(1)_, C_(2)_, C_(3)_, and C_(5)_ or conversaly oriented hydroxyl moieties are in C_(4)_ and C_(6)_.

Contrary to the rest of the tested unsubstituted carbohydrates, only β-D-allopyranose shows a *cis*-orientation of hydroxyl moieties in C_(1)_ and C_(5/6)_ in positions 2 and 6 of the tetrahydropyran ring together with the pyran oxygen in position 1 and *cis*-orientation of hydroxyl moieties in C_(2)_, C_(3)_ and C_(4)_ in positions 3, 4 and 5 of the tetrahydropyran ring, *i.e.*, *cis*-orientation of three sequential hydroxyl moieties. These facts are probably essential for interactions between risedronate monosodium salt and β-D-allopyranose. For example, α-D-galactopyranose possesses 1, 4, 5, 6 *cis*-oriented pyran oxygen together with hydroxyl moieties, α-D-glucopyranose possesses 1, 4, 6 *cis*-oriented pyran oxygen together with hydroxyl moiety, and α-D-mannopyranose possesses 3, 4, 6 *cis*-oriented hydroxyl moieties together with pyran oxygen in position 1.

According to the above mentioned hypothesis, interactions with risedronate should be predicted only for D-lyxofuranose from the furanose family. D-Lyxofuranose shows *cis*-orientation of hydroxyl moieties in C_(2)_ and C_(3)_ in positions 3 and 4 of the tetrahydrofuran ring together with furan oxygen in position 1. Nevertheless, this three-point interaction of D-lyxofuranose with risedronate is not sufficient for generation of a different form or co-crystal of risedronate. Only risedronate form H was generated using D-lyxofuranose. Probably the conformation of the tetrahydrofuran ring different from the pyranose chair conformation is also important.

Based on the screening of these unsubstituted carbohydrates and the above discussed hypothesis, it can be predicted that e.g., β-D-galactopyranose could be a successful candidate for modification of a crystalline form of risedronate. 

Different interactions of risedronate monosodium salt with phenyl-β-D-galactopyranoside compared to other evaluated *O*-substituted pyranosides are probably caused by the opposite orientation of hydroxyl moiety in C_(4)_ in position 5 of the tetrahydropyran ring. β-Position of the hydroxyl moiety in C_(1)_ of β-D-gluco- and β-D-galactopyranoside (as well as in β-D-allopyranose) possessing also *cis*-orientation to the pyran oxygen together with phenyl substitution of this hydroxyl moiety seem also to be important assumptions for interactions. For example, methyl-β-D-galactopyranoside did not show any interactions with risedronate, whereas phenyl-β-D-glucopyranoside and naphtyl-β-D-galactopyranoside generated H polymorph of risedronate, and phenyl-β-D-galactopyranoside provided a new form. Aliphatic alkoxy moieties (methoxy, octyloxy) show absolutely different physico-chemical properties, *i.e.*, non-binding interactions compared with the aromatic phenyl nucleus. On the other hand, a naphthyl moiety, which is comparable with a phenyl ring, does not meet steric requirements to generate a new form or entity with risedronate.

Contrary to the rest of the tested *O*-substituted pyranosides, phenyl-β-D-galactopyranoside shows *cis*-orientation of hydroxyl moieties in C_(3)_, C_(4)_ and C_(5/6)_ in positions 4, 5 and 6 of the tetrahydropyran ring, *i.e.*, three sequential hydroxyl moieties that possess *trans*-orientation with the phenoxy moiety in C_(1)_ in position 2 of the tetrahydropyran ring together with pyran oxygen in position 1. The hydroxyl moiety in C_(2)_ in position 3 of the tetrahydropyran ring possesses *cis*-orientation with the phenoxy moiety in C_(1)_ in position 2 of the tetrahydropyran ring and pyran oxygen in position 1. This configuration of all the hydroxyl moieties is probably essential for interactions between risedronate mono-sodium salt and phenyl-β-D-galactopyranoside.

Based on the screening of these carbohydrates, it was confirmed that the structure derived from β-D-galactopyranose could be a successful candidate for modification of a crystalline form of risedronate. It is also important to note that all the used carbohydrates can chelate the sodium cation in mono-sodium salt of risedronate, as discussed below.

### 2.2. Molecular Modelling

For better understanding of risedronate binding with β-D-allopyranose and phenyl-β-D-galacto-pyranoside, a molecular modelling of both complexes was carried out. Molecular Dynamics (MD) simulates the behaviour of studied molecules in a given force field using Newton's equation of motion. In this way an ensemble of successive states is generated, and molecule properties are predicted by system calculations. So, in this particular case, the behaviour of the complexes and interaction between risedronate and β-D-allopyranose or risedronate and phenyl-β-D-galactopyranoside were described by molecular dynamic simulation.

**Figure 18:**
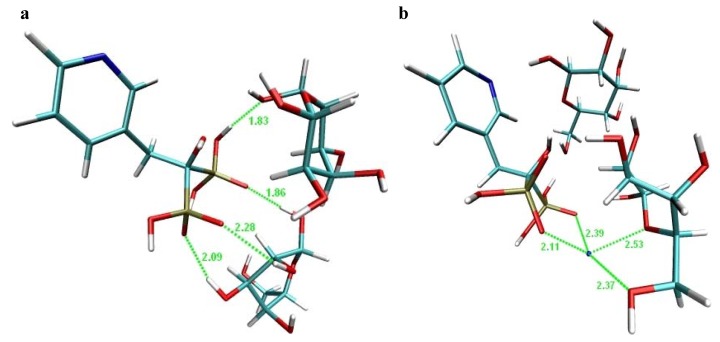
Illustration of supposed interactions of risedronate mono-sodium salt with β-D-allopyranose: **(a)** suggested interaction between molecule of risedronate and allose; **(b)** suggested interaction between sodium cation and allose.

**Figure 19:**
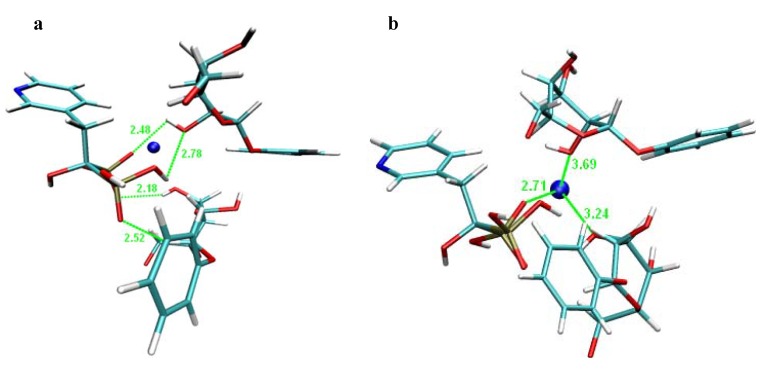
Illustration of supposed interactions of risedronate mono-sodium salt with phenyl-β-D-galactopyranoside: **(a)** suggested interaction between molecule of risedronate and phenyl-β-D-galactopyranoside; **(b)** suggested interaction between sodium cation and phenyl-β-D-galactopyranoside.

The structures illustrated in Figure 18 and Figure 19 can be understood as two possible extremes of a risedronate-allose (Figure 18) and risedronate-phenylgalactopyranoside (Figure 19) complex formation. The MD showed that the structure of risedronate allowed forming a complex with β-D-allopyranose (Figure 18a) or phenyl-β-D-galactopyranoside (Figure 19a). Interactions between the sodium cation and β-D-allopyranose are illustrated in Figure 18b, and interactions between the sodium cation and phenyl-β-D-galactopyranoside, in Figure 19b. As both β-D-allose and phenyl-β-D-galactopyranoside are not detectable in the prepared crystalline forms (all figures probably show only intermediates), both carbohydrates modify the environment from which risedronate crystallizes under kinetically (in case of allose) and thermodynamically (in case of phenylgalactopyranoside) controlled conditions of crystallization, when methanol is used as anti-solvent by binding allose or phenylgalactopyranoside hydroxyl moieties to risedronate phosphonate groups and/or chelation of the sodium cation. All illustrated complexes probably disintegrate, and risedronate mono-sodium salt crystallizes alone without counterions in a different crystalline form.

### 2.3. In vitro Screening of Absorption (PAMPA experiments)

Many low-molecular-weight drugs are absorbed through passive (or partially passive) transport. The Parallel Artificial Membrane Permeability Assays (PAMPA) have become a very useful and quite cheap tool for predicting *in vivo* drug permeability and are well-suited as a ranking tool for the assessment of compounds with passive transport mechanisms. An absorption study of binary mixtures or final formulations is also possible on PAMPA plates. PAMPA can be used as an alternative approach to assess *in vitro* transcellular passive permeation [17]. As sample **6** was not detected as potential co-crystals, penetration experiments were not performed.

## 3. Experimental

### 3.1. General

All reagents, excipients and solvents of analytical grade were purchased from Sigma-Aldrich. Semi-crystalline risedronate monosodium salt used as a starting material is a product of Zentiva k.s. [22]. Near infrared spectra were recorded using a Smart Near-IR UpDrift™, Nicolet™ 6700 FT-IR Spectrometer (Thermo Scientific, USA). The spectra were obtained by accumulation of 128 scans with 4 cm^–1^ resolution in the region of 12,800–4,000 cm^−1^. FT-Raman spectra were accumulated by an FT-Raman spectrometer RFS 100/S (Karlsruhe, Bruker, Germany). The spectra were obtained by accumulation of 256 scans with 4 cm^−1^ resolution in the back scattering geometry with the laser wavelength of 1064 nm. ^31^P CP/MAS NMR Spectra were recorded on a Bruker AVANCE 500 MHz spectrometer (Karlsruhe, Bruker, Germany). The ^31^P CP/MAS spectra were measured in 4 mm rotor at 10 kHz with 2 ms contact time. ^31^P chemical shift of NH_4_H_2_PO_4_ (0 ppm) was used as an external reference for ^31^P chemical shift. The ^13^C CP/MAS spectra were measured in 4 mm rotor at 13 kHz with 2 ms contact time. Carbon chemical shifts were referenced to the signal for TMS via a replacement sample of glycine (176 ppm for the carbonyl group signal). The XRPD patterns were obtained on a PANalytical X’PERT PRO MPD diffractometer with Cu Kα radiation (45 kV, 40 mA). The powder samples were measured on Silica plate holder. Data were recorded in the range 2-40° 2θ, with 0.01° 2θ step size and 50s/step scan speed. For the measurement of differential scanning calorimetry (DSC) curve an instrument DSC Pyris 1 (PerkinElmer, USA) was used. Maximum sample weight was 3.5 mg, and the standard Al sample pan was used. The record of the DSC curve was in the range of 50–300 °C at the rate of 10.0 °C/min under a nitrogen atmosphere.

### 3.2. Generation of Sampless

All the evaluated samples with ratios 1:1, 1:2 and 1:3 were prepared by means of dissolution of semi-crystalline risedronate monosodium salt and the excipient in water, subsequently mixed (1 h) and slowly evaporated at ambient temperature. To some samples with ratios 1:2 and 1:3 methanol (5 mL) was slowly added dropwise as an anti-solvent. The solid precipitated compound was filtered and dried at ambient temperature and the remaining liquid part was slowly evaporated at ambient temperature. All generated solid compounds were subsequently screened by means of FT-NIR and FT-Raman spectroscopy. If a sample differing from the starting materials was found, it was additionally characterized using the above mentioned solid state analytical techniques. Particular preparations of the risedronate and phenyl-β-D-galactopyranoside samples in various ratios are described in Table 1 and Table 2.

**Table 1:** Risedronate (RSN) and phenyl-β-D-galactopyranoside (Ph-gal) in ratios 1:1, 1:2 and 1:3 (samples **1**-**3**).

Comp.	1:1		1:2		1:3	
	Amount [g]	Water [mL]	Amount [g]	Water [mL]	Amount [g]	Water [mL]
**RSN**	0.6017	10	0.6010	10	0.6007	10
**Phe-gal**	0.5054	2.0	1.0095	3.0	1.5136	3.5

**Table 2:** Risedronate (RSN) and phenyl-β-D-galactopyranoside (Ph-gal) in ratios 1:2 and 1:3 with addition of methanol (samples **4**-**7**).

Comp.	1:2			1:3		
	Amount [g]	Water [mL]	MeOH [mL]	Amount [g]	Water [mL]	MeOH [mL]
**RSN**	0.6015	10	5.0	0.6012	10	5.0
**Phe-gal**	1.0104	3.0	5.0	1.5149	3.5	5.0

### 3.3. Molecular Modelling

Both of the starting structures, risedronate and β-D-allopyranose, were built by Avogadro software [30] and minimized at a MMFF94 force field level. The geometry of the systems was optimized by Gaussian 03 [31] using HF/6-31G*. The ESP (electrostatic potential) charges were calculated at the same level HF/6-31G*. The preparation of the system for molecular dynamics was performed using the Leap module of the Amber package, GAFF force field [32].

## 4. Conclusions

Twelve sugar alcohols, furanoses, pyranoses and eight gluco-, manno- and galactopyranoside derivatives were tested as counterions for generation of co-crystals with risedronate monosodium salt. One hundred and fifty-two samples were prepared. All samples were screened by FT-NIR and FT-Raman spectroscopy. NIR spectra of the prepared samples were compared with the starting materials, the subtraction results of the samples and the starting carbohydrates were calculated, and a different form of risedronate only in the presence of phenyl-β-D-galactopyranoside was predicted and checked by spectroscopy and ^31^P CP/MAS NMR spectroscopy. Using ^13^C CP/MAS NMR spectroscopy, XRPD and DSC, it was clearly proved that it is not possible to detect phenyl-β-D-galactopyranoside in the sample, and according to the XRPD pattern, a new solid phase was formed and named as polymorph P. In case of used β-D-allopyranose only the impure form B of risedronate was generated. No co-crystal was prepared, therefore PAMPA penetration experiments were not performed. It can be stated that allose and especially phenyl-β-D-galactopyranoside modifies the environment from which risedronate is crystallized under kinetically (in case of allose) or thermodynamically (in case of phenylgalactopyranoside) controlled conditions of crystallization. Phenyl-β-D-galactopyranoside caused new solid phase risedronate monosodium salt formation due to unique orientation of hydroxyl moieties in the tetrahydropyran ring together with the phenoxy moiety in C_(1)_ in positions 2 of the tetrahydropyran ring (similarly β-D-allopyranose induced formation of risedronate polymorph B due to specific orientation of hydroxyl moieties). The MD simulation revealed that the structure of risedronate allowed forming a complex with phenyl-β-D-galactopyranoside and β-D-allopyranose. Furthermore the sodium cation can contribute to the binding of risedronate and β-D-allopyranose or phenyl-β-D-galactopyranoside. The sodium cation makes this complex energetically favourable and helps to retain the proper topology of the binding phosphates of risedronate and the proper orientation of the hydroxyl moieties of β-D-allopyranose or phenyl-β-D-galactopyranoside.
